# Human amnion-derived mesenchymal stem cells promote osteogenic differentiation of human bone marrow mesenchymal stem cells via H19/miR-675/APC axis

**DOI:** 10.18632/aging.103277

**Published:** 2020-05-20

**Authors:** Xiaojie Ma, Yifeng Bian, Hua Yuan, Ning Chen, Yongchu Pan, Weina Zhou, Shiyu Gao, Xin Du, Shushu Hao, Zixin Yan, Xuan Li, Keyue Liu, Fan Xu, Yuli Wang, Yifei Du

**Affiliations:** 1 Jiangsu Key Laboratory of Oral Diseases, Nanjing Medical University, Nanjing, China; 2 Department of Oral and Maxillofacial Surgery, Affiliated Hospital of Stomatology, Nanjing Medical University, Nanjing, China; 3 Department of Orthodontics, Affiliated Hospital of Stomatology, Nanjing Medical University, Nanjing, China; 4 Department of Temporomandibular Joint, Affiliated Hospital of Stomatology, Nanjing Medical University, Nanjing, China; 5 State Key Laboratory of Bioelectronics, Southeast University, Nanjing, China

**Keywords:** human amnion-derived mesenchymal stem cells, osteogenic differentiation, long noncoding RNA H19, miR-675, adenomatous polyposis coli

## Abstract

Bone volume inadequacy is an emerging clinical problem impairing the feasibility and longevity of dental implants. Human bone marrow mesenchymal stem cells (HBMSCs) have been widely used in bone remodeling and regeneration. This study examined the effect of long noncoding RNAs (lncRNAs)-H19 on the human amnion-derived mesenchymal stem cells (HAMSCs)-droved osteogenesis in HBMSCs. HAMSCs and HBMSCs were isolated from abandoned amniotic membrane samples and bone marrow. The coculture system was conducted using transwells, and H19 level was measured by quantitative real-time reverse transcription-polymerase chain reaction (RT-PCR). The mechanism was further verified. We here discovered that osteogenesis of HBMSCs was induced by HAMSCs, while H19 level in HAMSCs was increased during coculturing. H19 had no significant effect on the proliferative behaviors of HBMSCs, while its overexpression of H19 in HAMSCs led to the upregulated osteogenesis of HBMSCs *in vivo* and *in vitro*; whereas its knockdown reversed these effects. Mechanistically, H19 promoted miR-675 expression and contributed to the competitively bounding of miR-675 and Adenomatous polyposis coli (APC), thus significantly activating the Wnt/β-catenin pathway. The results suggested that HAMSCs promote osteogenic differentiation of HBMSCs via H19/miR-675/APC pathway, and supply a potential target for the therapeutic treatment of bone-destructive diseases.

## INTRODUCTION

Bone volume inadequacy is a crucial problem among aged patients requiring dental implants. Mesenchymal stem cells (MSCs) possess multi-potentiality and self-renewal properties, which have been extensively applied in the treatment of different degenerative conditions, including bone defects [1]. Human bone marrow mesenchymal stem cells (HBMSCs) are the best characterized multipotent adult stem cells; HBMSCs possess self-renewal capacity, low anti-inflammatory properties, and less risk of malignant transformation during *in vitro* amplification [2]. However, HBMSCs also have various disadvantages, such as high traumatic response, limited availability and reduced stemness during ageing [3]. Some pathological status also has direct detrimental effects on HBMSCs, which remarkably influence the cell retention and survival at the target region. Studies have suggested that human amnion-derived mesenchymal stem cells (HAMSCs) may be characterized by MSCs features, but also show embryonic stem cells-like phenotypic characteristics [4]. Besides, the process of isolation of HAMSCs from the abandoned amniotic membrane is considered very safe, noninvasive, and ethical [5]. Our previous research has shown that HBMSCs may be differentiated to osteoblast lineage when co-culturing with HAMSCs [6]. Therefore, HAMSCs could be regarded as an alternative method for bone regeneration.

Long non-coding RNAs (lncRNAs) are a class of non-coding RNAs with about 200-300 long nucleotides, which activate the transcription and post-transcription levels [7]. lncRNAs are involved in many biological and pathological processes, including cellular progression, differentiation, carcinogenesis, and chronic diseases [8]. Osteogenesis-related lncRNAs exert their biological functions by activating multiple molecules, while they also have a unique role in the osteogenic differentiation of various types of cells [9]. For example, studies have reported that lncRNA H19 has an important role in activating osteogenic differentiation as a highly conserved noncoding transcript with a shallow mutation rate during evolution [10, 11]. In this study, we investigated the roles of H19 in HAMSCs-droved osteogenic differentiation.

MicroRNAs (miRNAs) are a class of small non-coding RNAs about 18–24 nucleotides long that activate gene expression at a posttranscriptional level by binding to the (3’ UTR) of the target mRNAs, and subsequently causing mRNA repression or activation [12]. Several miRNAs have been proved to be involved in the process of osteogenesis [13, 14]. Previous studies have shown that LncRNA could serve as a primary miRNA precursor or competing endogenous RNA, thus acquiring functionality and influencing target gene expression [15, 16]. H19 is a primary miRNA precursor for microRNA-675 (miR-675) and the H19/miR-675 axis has been found in multiple biological processes, such as diabetic cardiomyopathy and tumorigenesis [17, 18]. Despite the previous achievement, the role of the H19/miR-675 axis in the HAMSCs-droved osteogenic differentiation remains unknown. In this paper, we explored whether H19 promotes the HAMSCs-droved osteogenic differentiation while miR-675 increased. Moreover, miR-675 performed its inhibitory effect on Adenomatous polyposis coli (APC), an inhibitor of β-catenin [19], thus inducing β-catenin translocate to the nucleus and activating Wnt/β-catenin signaling. This study provides references for the lncRNA-miRNA-mRNA analysis and proposes a therapeutic target for the treatment of bone deficiency.

## RESULTS

### LncRNA-H19 expression in HAMSCs increases with the HAMSCs-droved osteogenesis

**Figure 1:**
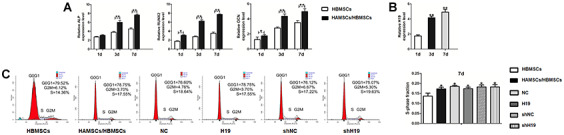
**Osteogenic differentiation of HBMSCs cocultured with HAMSCs, lncRNA-H19 expression in HAMSCs and effects of lncRNA-H19 in HAMSCs on the proliferation of HBMSCs.** (**A**) Relative mRNA expressions of ALP, RUNX2 and OCN in HBMSCs cocultured with HAMSCs were measured by RT-PCR analysis. (**B**) LncRNA-H19 expression in HAMSCs during coculturing was measured by RT-PCR analysis. (**C**) HBMSCs proliferation was demonstrated by flow cytometry. Data are shown as mean ± SD. *P < 0.05 and **P < 0.01.

Expression level of H19 was detected and the stably expressing cells (HAMSCs^NC^, HAMSCs^H19^, HAMSCs^shNC^ and HAMSCs^shH19^) were sorted for subsequent experiments (Supplementary Figure 1A and 1B). Previous studies have indicated that HAMSCs stimulates osteogenic differentiation of HBMSCs [20]. In order to verify these findings, we built a transwell co-culture model of HAMSCs/HBMSCs and examined the expression of early- and late-stage osteogenic markers. Compared with the HBMSCs group, 1, 3 and 7 days of HAMSCs coculturing gradually upregulated the mRNA expressions of ALP, RUNX2 (early-stage osteogenic markers) and OCN (late-stage marker) in HBMSCs (Figure 1A). Likewise, RNA samples derived from HAMSCs expressed significantly increased levels of H19 in a time-dependent manner along with the osteogenic differentiation of HBMSCs (Figure 1B).

### LncRNA-H19 expression in HAMSCs has no effects on HBMSCs proliferation

To examine the effects of lncRNA-H19 in HAMSCs on HBMSCs proliferation, lentivirus containing H19 was transfected in HAMSCs. Flow cytometry analysis revealed distinct differences in S-phase checkpoints between HBMSCs group and HAMSCs/HBMSCs group. On the other hand, no significant difference was found in the proliferative index among HAMSCs/HBMSCs, NC, H19, shNC and shH19 group (Figure 1C), which further suggests that lncRNA-H19 in HAMSCs does not increase HBMSCs proliferation in HAMSCs/HBMSCs coculture system.

### LncRNA-H19 in HAMSCs promotes osteogenesis of HBMSCs

**Figure 2:**
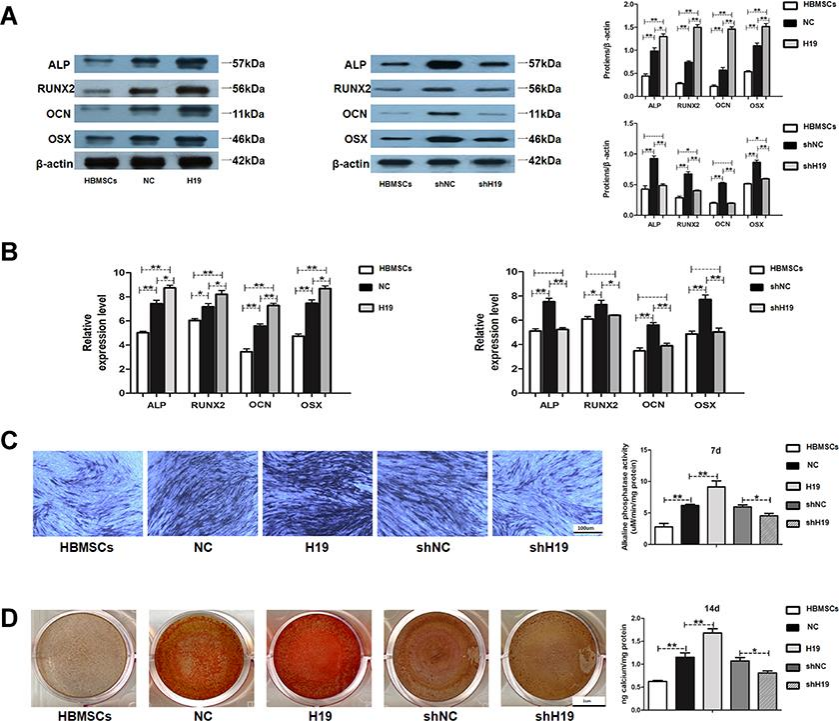
**LncRNA-H19 in HAMSCs promotes osteogenic differentiation of HBMSCs.** (**A**) Protein levels of ALP, RUNX2, OCN, and OSX were assessed by western blot assay in HBMSCs, NC, H19, shNC and shH19 groups. (**B**) Relative mRNA expressions of ALP, RUNX2, OCN and OSX were measured by RT-PCR analysis in HBMSCs, NC, H19, shNC and shH19 groups. (**C**) ALP staining and activity in HBMSCs, NC, H19, shNC, and shH19 groups. Scale bar, 100 μm. (**D**) Alizarin red staining and quantification in HBMSCs, NC, H19, shNC, and shH19 groups. Scale bar, 1cm. Data are shown as mean ± SD. *P < 0.05 and **P < 0.01.

HAMSCs transfected with lentivirus were coclutured with HBMSCs and induced in osteoblast differentiation medium. Stably transfected cells were assigned into NC: HAMSCs^NC^/HBMSCs, H19:HAMSCs^H19^/HBMSCs, shNC: HAMSCs^shNC^/HBMSCs and shH19:HAMSCs^shH19^/HBMSCs. The effect of H19 in HAMSCs on the osteogenic differentiation of HBMSCs was further examined. Western blot assay showed that the protein levels of ALP, RUNX2, OCN and OSX were markedly higher in H19 group compared with those in HBMSCs and NC groups, whereas the H19 knockdown reversed the positive effects of HAMSCs (Figure 2A). As shown by RT-PCR, the mRNA levels of ALP, RUNX2, OCN, and OSX were increased by H19 overexpression, whereas H19 knockdown obtained the opposite effects in shH19 group (Figure 2B). The ALP staining and activity were enhanced in the H19 group and decreased by H19 knockdown (Figure 2C). Besides, Alizarin red staining and quantification showed upregulated matrix mineralization in the H19 group compared with those in HBMSCs and NC groups, whereas H19 knockdown showed the opposite effects (Figure 2D). These results indicated that H19 in HAMSCs promotes osteogenesis of HBMSCs.

**Figure 3:**
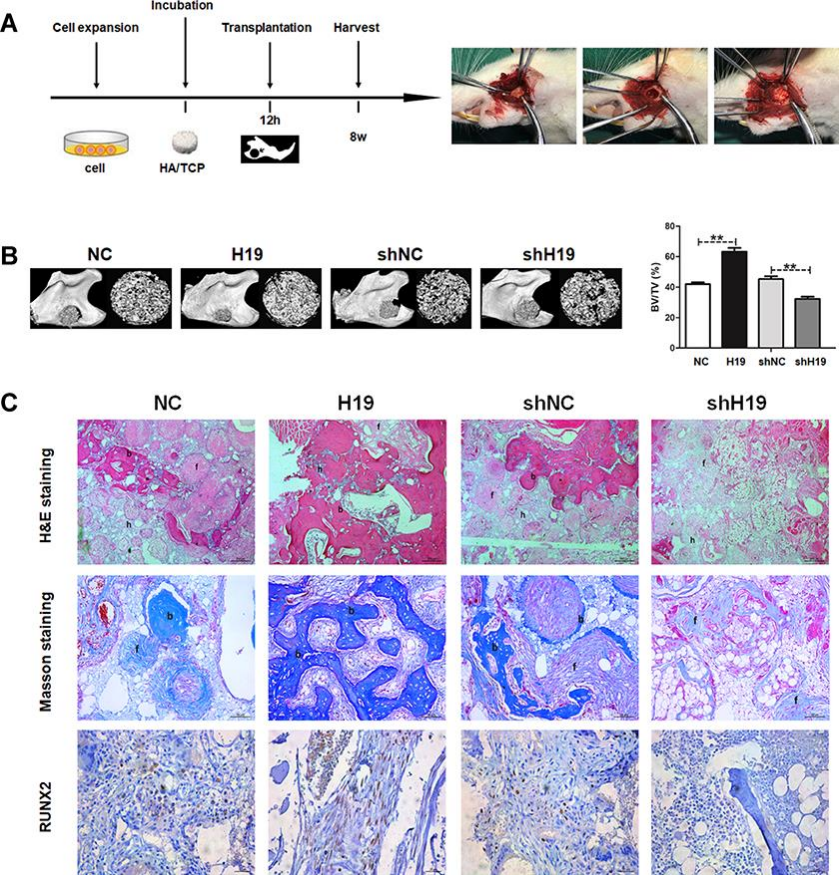
**LncRNA-H19 in HAMSCs promotes osteogenesis *in vivo*.** (**A**) NC, H19, shNC and shH19 groups were transplanted subcutaneously into a rat critical-sized mandibular defect model for 8 weeks. (**B**) Reconstructed 3D micro-CT images of the tissue-engineered bone and percentages of BV/TV. (**C**) H&E staining, Masson staining and immunohistochemical staining of RUNX2 in NC, H19, shNC and shH19 groups. b: bone-like tissues, h: HA/TCP scaffold, f: fibrous. Scale bar, 200 μm. Data are shown as mean ± SD. **P < 0.01.

Next, we examined the effect of H19 on *in vivo* bone formation in a rat critical-sized mandibular defect model (4 rats in each group) for 8-week growth (Figure 3A). The results were expressed as a percentage of mineralized volume fraction (bone volume/total volume, BV/TV). H19-overexpressing increased BV/TV compared with the NC group, while a significant decreased BV/TV was detected in the shH19 group compared with shNC group (Figure 3B). Histological examination by H&E and Masson staining was consistent with the results of BV/TV. More organized bone matrix was formed in the H19 group compared to the NC group, whereas there was a significant amount of fibrous tissue in the shH19 group compared with shNC group. Furthermore, the abundance of RUNX2 was upregulated in the H19 group compared to NC group and downregulated in the shH19 group compared to shNC group, which was confirmed by immunohistochemistry (Figure 3C).

### MiR-675 serves as a downstream of LncRNA-H19 in HAMSCs-droved osteogenesis

**Figure 4:**
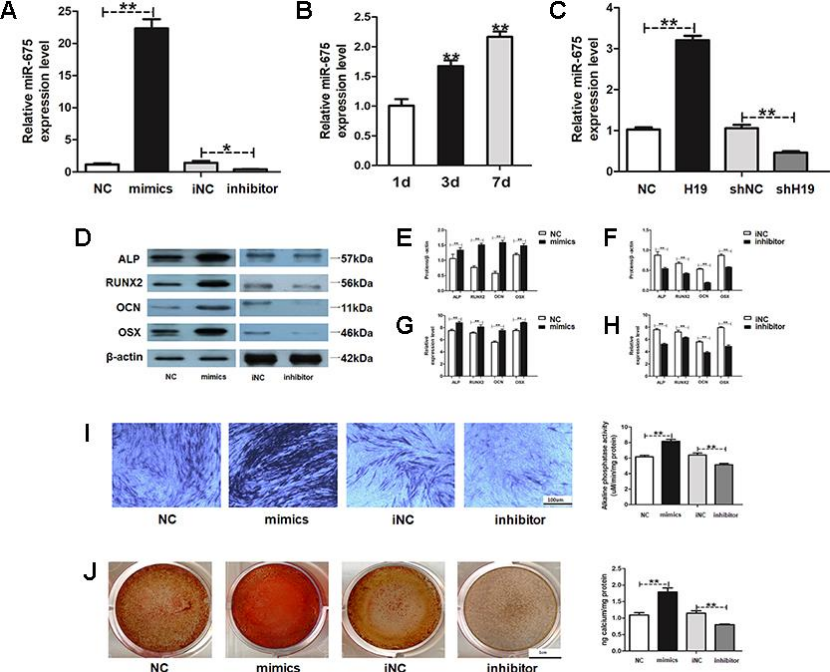
**MiR-675 in HAMSCs is activated by lncRNA-H19 and promotes osteogenic differentiation of HBMSCs.** (**A**) Transfection efficacy of miR-675 was detected by RT-PCR. (**B**) MiR-675 expression in HAMSCs during coculturing was measured by RT-PCR. (C) MiR-675 expression was measured by RT-PCR in NC, H19, shNC, and shH19 groups. (**D**–**F**) Protein levels of ALP, RUNX2, OCN and OSX were assessed by western blot assay in NC, mimics, iNC and inhibitor groups. (**G**, **H**) Relative mRNA expressions of ALP, RUNX2, OCN, and OSX were measured by RT-PCR analysis in NC, mimics, iNC and inhibitor groups. (**I**) ALP staining and activity in NC, mimics, iNC and inhibitor groups. Scale bar, 100 μm. (**J**) Alizarin red staining and quantification in NC, mimics, iNC and inhibitor groups. Scale bar, 1 cm. Data are shown as mean ± SD. *P < 0.05 and **P < 0.01.

To investigate how H19 promotes the HAMSCs-droved osteogenesis, miR-675, whose primary precursor is H19, was determined. The transfection efficacy of miR-675 was detected by RT-PCR (Figure 4A). Along with H19 level, 1, 3 and 7 days of coculturing upregulated the expression of miR-675 in HAMSCs in a time-dependent manner (Figure 4B). Meanwhile, the expression of miR-675 was increased by H19 overexpression, whereas the H19 knockdown showed the opposite effects in the shH19 group (Figure 4C). After that, miR-675 mimics and inhibitor were used to transiently transfect HAMSCs. Stably transfected cells were assigned into NC: HAMSCs ^miR-675 NC^/HBMSCs, mimics: HAMSCs ^miR-675 mimics^/HBMSCs, iNC: HAMSCs ^miR-675 iNC^/HBMSCs and inhibitor: HAMSCs ^miR-675 inhibitor^/HBMSCs. Western blot assay showed that several osteogenic marker proteins were markedly higher in mimics group compared with those in NC groups, whereas the miR-675 knockdown obtained opposite effects (Figure 4D–4F). In addition, mRNA levels of several osteogenic marker genes were increased by miR-675 overexpression and decreased when miR-675 knockdown (Figure 4G and 4H). Furthermore, ALP staining and activity were enhanced in the mimics group and decreased in the inhibitor group (Figure 4I). Moreover, Alizarin red staining and quantification also confirmed that overexpression of miR-675 led to increased calcified nodules, while miR-675 knockdown alleviated the effects in the committed differentiation of HAMSCs/HBMSCs (Figure 4J). Collectively, the above findings indicated that miR-675 in HAMSCs serves as a downstream of LncRNA-H19 and promotes osteogenesis of HBMSCs.

### MiR-675 targets APC and downregulates APC expression in HAMSCs

**Figure 5:**
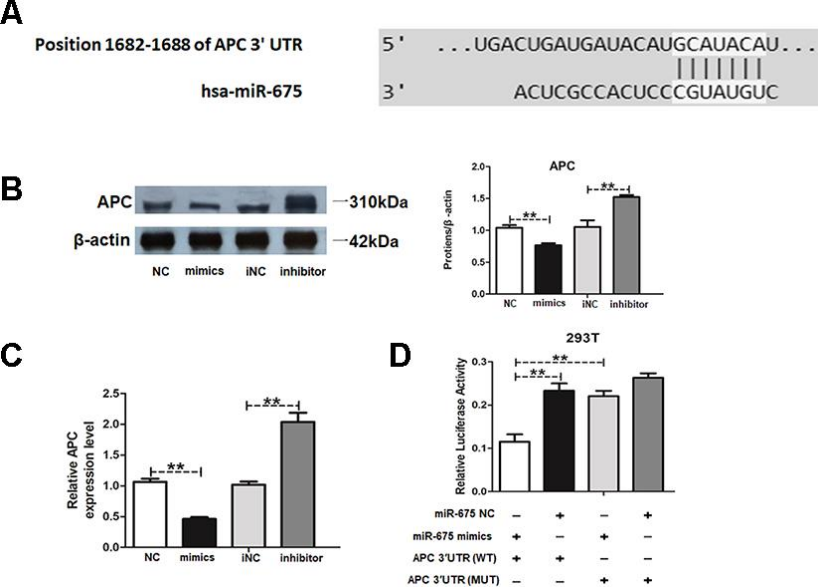
**MiR-675 downregulates APC expression in HAMSCs.** (**A**) The potential binding sites between APC and miR-675 predicted by biological software. (**B**) Protein level of APC was assessed by western blot assay in NC, mimics, iNC and inhibitor groups. (**C**) Relative mRNA expression of APC was measured by RT-PCR analysis in NC, mimics, iNC and inhibitor groups. (**D**) Luciferase reporter assay was used to validate the target in 293T cells. Relative Renilla luciferase activity was normalized to that of firefly luciferase. Data are shown as mean ± SD. **P < 0.01.

To further explore the mechanisms of miR-675 in HAMSCs promoting osteogenesis of HBMSCs, the candidate target gene was searched by Target Scan software. APC was predicted as the potential target of miR-675 (Figure 5A). APC is a negative regulator in the activation of Wnt/β-catenin pathway, which is closely related to the osteogenesis of MSCs [21, 22]. Western blot results proved that APC was significantly decreased by miR-675 mimics and increased by miR-675 inhibitor. RT-PCR also revealed that miR-675 suppressed mRNA levels of APC in HAMSCs (Figure 5B and 5C). Luciferase activity of APC wild-type reporter was successfully reduced by miR-675 mimics, and mutation of the putative miR-675 target sites rescued the previous suppressive effect (Figure 5D). These findings confirmed the binding condition between miR-675 and APC, thus suggesting that APC is a direct target of miR-675.

### APC overexpression inhibited Wnt/β-catenin pathway in HAMSCs and alleviated HAMSCs-droved osteogenesis in HBMSCs

**Figure 6:**
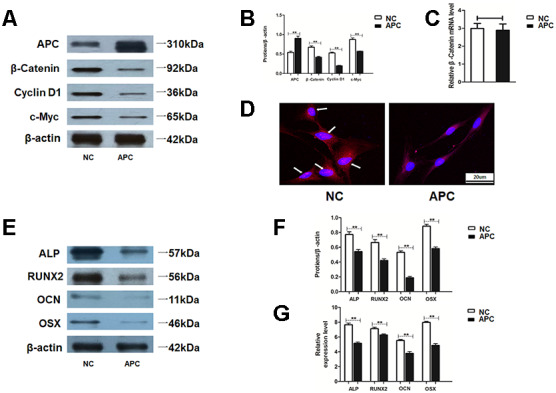
**APC inhibits Wnt/β-catenin pathway and HAMSCs-droved osteogenesis.** (**A**, **B**) Protein levels of β-catenin, Cyclin D1 and c-Myc were assessed by western blot assay in NC and APC groups. (**C**) Relative mRNA expression of β-catenin was measured by RT-PCR analysis in NC and APC groups. (**D**) Immunofluorescence staining showed the β-catenin location in NC and APC groups. Scale bar, 20 μm. (**E**, **F**) Protein levels of ALP, RUNX2, OCN and OSX were assessed by western blot assay in NC and APC groups. (**G**) Relative mRNA expressions of ALP, RUNX2, OCN, and OSX were measured by RT-PCR analysis in NC and APC groups. Data are shown as mean ± SD. **P < 0.01.

The influence of APC in HAMSCs was further examined. Western blot assay determined proteins related to the Wnt/β-catenin pathway, including β-catenin, Cyclin D1 and c-Myc, were remarkably downregulated by APC overexpression in HAMSCs (Figure 6A and 6B). Interestingly, the mRNA level of β-catenin showed no difference between the NC group and the APC group (Figure 6C). These results suggested that APC overexpression promoted β-catenin protein degradation, while the mRNA level of β-catenin was not influenced. Moreover, immunofluorescence staining showed that APC overexpression induced a reduction in nuclear β-catenin accumulation and strongly decreased the nuclear β-catenin protein level (Figure 6D). The above findings indicated that APC overexpression inhibited Wnt/β-catenin pathway in HAMSCs.

To further explore whether the pro-osteogenic effect of HAMSCs was inhibited by APC, we assessed the committed differentiation of HBMSCs cocultured with HAMSCs. Western blot assay showed that several osteogenic marker proteins in HBMSCs were markedly higher in the NC group compared with those in APC groups. In addition, mRNA levels of several osteogenic marker genes in HBMSCs were decreased by APC overexpression in HAMSCs (Figure 6E–6G). In a word, APC overexpression in HAMSCs could reverse HAMSCs-droved osteogenesis.

### MiR-675 mimic can rescue the APC overexpression and osteogenesis deficiency caused by shH19 in HAMSCs

**Figure 7:**
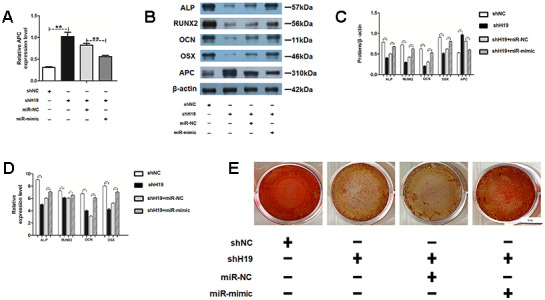
**MiR-675 mimic could rescue the shH19 mediated inhibitory effects.** (**A**) Relative mRNA expression of APC was measured by RT-PCR analysis. (**B**, **C**) Protein levels of ALP, RUNX2, OCN, OSX, and APC were assessed by western blot assay. (**D**) Relative mRNA expressions of ALP, RUNX2, OCN, and OSX were measured by RT-PCR analysis. (**E**) Alizarin red staining analysis. Scale bar, 1 cm. Data are shown as mean ± SD. *P < 0.05 and **P < 0.01.

**Figure 8:**
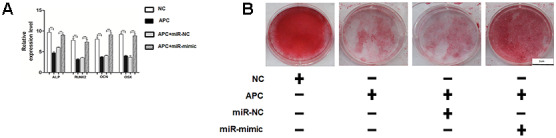
**MiR-675 mimic could rescue the APC overexpression mediated inhibitory effects.** (**A**) Relative mRNA expressions of ALP, RUNX2, OCN, and OSX were measured by RT-PCR analysis. (**B**) Alizarin red staining analysis. Scale bar, 1 cm. Data are shown as mean ± SD. **P < 0.01.

**Figure 9:**
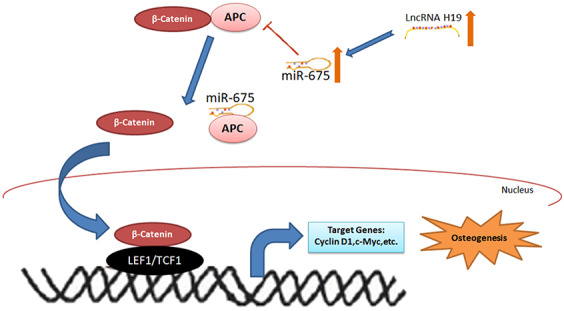
**The schematic diagram for lncRNA-H19/miR-675/APC/Wnt/β-catenin axis in this study.**

To further determine the roles of miR-675 and APC involved in H19-mediated osteogenesis, the rescue assays were carried out. RT-PCR suggested that co-transfection with miR-675 mimicked and shH19 rectified APC overexpression compared to the shH19 group (Figure 7A). As shown in Figure 7B–7E, shH19-mediated osteogenesis suppression could also be rescued in co-transfected cells. Moreover, RT-PCR and Alizarin red staining suggested that MiR-675 mimic could rescue the APC overexpression mediated inhibitory effects (Figure 8A, 8B). Taken together, lncRNA-H19 increases the miR-675 level, targets APC transcription, and activates the Wnt/β-catenin pathway to promote the HAMSCs-droved osteogenic differentiation (Figure 9).

## DISCUSSION

As a significant type of MSCs, HAMSCs derived from amniotic membrane have fewer ethical concerns [23]. Our recent studies have confirmed that HAMSCs are capable of driving osteogenesis of HBMSCs under physiological and pathological conditions [6, 24, 25]. LncRNAs, whose transcripts are over 200 nucleotides in length, are known to be involved in the multilineage differentiation of MSCs at the transcriptional and post-transcriptional levels [26–29]. LncRNA-H19 abundantly conserves non-coding transcripts, expresses very low mutation rate during fetal life, and participates in multiple biological processes [11, 30]. Hence, we examined the relationship between H19 and HAMSCs-droved osteogenesis.

We demonstrated that levels of H19 in HAMSCs increased along with the osteogenic differentiation in HBMSCs cocultured with HAMSCs. The regulatory proliferation of H19 has been widely reported, albeit with some inconsistencies in the observed results [31, 32]. In this study, the proliferative rate of HBMSCs was enhanced by HAMSCs, while we did not observe the influence of H19 in HAMSCs on HBMSCs proliferation. These results suggest that the underlying mechanism through which H19 activates cell proliferation is complicated.

Next, we identified the influence of H19 in HAMSCs on HBMSCs osteogenic differentiation by rescue experiments *in vitro*. ALP represents an early marker of osteogenic differentiation in the initial stage of calcification [33]. RUNX2 has a primary function in bone tissue enrichment, where deficiency directly suppresses osteogenic specificity [34]. OSX, as the downstream of RUNX2, is closely related to morphogenesis and has great importance in bone regeneration [35]. OCN is primarily generated in the late stage of osteoblast differentiation, and therefore its expression could specifically display bone formation [34]. We found that the above osteogenic markers (ALP, RUNX2, OCN, and OSX) was upregulated by H19 overexpression and downregulated by H19 knockdown. Besides, the promotion of osteoblast differentiation was detected in the H19 group, whereas the shH19 group showed the opposite effects. Moreover, *in vivo* study also demonstrated that H19-overexpressing HAMSCs induced bone formation and osteoblastic activity.

H19 is a paternally imprinted gene that does not encode a protein, but rather a 2.3-kb H19 ncRNA [36]. Moreover, H19 performs a valuable biological function with a very low mutation rate in exons [10]. For instance, H19 has been identified as a key promotor associated with bone formation [37, 38]. In addition, it has been reported that H19 post-translationally promotes different biological processes by increasing the expression of miR-675 as its precursor [17, 39, 40]. MiR-675 is partially responsible for the pro-osteogenic activity of H19 and enhances bone development in MSCs [11]. We found that the expression of miR-675 in HAMSCs increased in a time-dependent manner along with the H19 level. Thus, the critical roles of miR-675 in the underlying pro-oncogenic mechanism were further explored. Initially, we demonstrated that miR-675 expression was positively increased by H19. Secondly, our results verified that during coculturing, miR-675 in HAMSCs positively promoted HBMSCs osteogenesis. In addition, the promotion of osteoblast differentiation was observed in miR-675 mimics group, whereas miR-675 inhibitor group showed the opposite effects.

H19/miR-675 was proved to promote osteogenic differentiation by targeting several transforming growth factors in the TGF-βsignaling pathway [41]. Interestingly, other pathways play critical roles in cellular differentiation were not deeply investigated. Wnt/β-catenin pathway could be activated by a series of external stimuli, exerting a vital role in cellular behaviors [42, 43]. The nuclear localization of β-catenin intuitively reflects the activation level of Wnt/β-catenin pathway [44]. Notably, negative regulators such as GSK3β and APC target β-catenin as a protein complex, and directly inhibit β-catenin transferring into nuclear [45]. To explore the underlying mechanism of H19/ miR-675 axis, bioinformatics analysis, and luciferase activity assay were carried out. The obtained results revealed that miR-675 can directly bind to APC. Hence, we hypothesized that lncRNA-H19 increased miR-675, contributed to the competed sponging of miR-675 and APC, then decreased APC and released β-catenin into nuclear. To validate our hypothesis, we first performed western blot and RT-PCR, which revealed that APC was significantly decreased by miR-675 mimics and increased by miR-675 inhibitor. Second, we identified the relationship between the Wnt/β-catenin pathway and APC. As expected, a significant reduction in nuclear β-catenin accumulation was detected by APC overexpression. Moreover, we also confirmed that APC overexpression in HAMSCs could reverse HAMSCs-droved osteogenesis in HBMSCs. The rescue assays showed that co-transfection with miR-675 mimic and shH19 rectified APC overexpression and rescued shH19-mediated osteogenesis suppression. Together, these results indicated that lncRNA-H19 epigenetically inhibited SPAG9 transcription via miR-675 generation.

The interaction between HAMSCs and HBMSCs might through multiple mechanisms, including growth factors, cytokines and exosomes. MSCs commonly secret numerous soluble growth factors and cytokines associated with angiogenesis, osteogenesis, chemotaxis and inflammation during incubation [46, 47]. Those growth factors and cytokines might be responsible for the signaling pathway transferring between two cells. One the other hand, exosomes secreted by mammalian cells are also potential medium. Exosomes are membrane-bound phospholipid vesicles (40–150 nm in diameter) of endocytic origin [48]. These vesicles may contain proteins, mRNAs, non-coding RNAs and other specific cargo [49, 50]. Upon secretion into the extracellular environment, exosomes have been demonstrated to carry their cargo to target cells, protecting the cargo from degradation during transportation [51]. Therefore, the H19/miR-675/APC axis in HAMSCs might be carried by exosomes and transferred to HBMSCs, which then promotes osteogenic differentiation. The detailed feedback loops in HBMSCs coclutured with HAMSCs need to be investigated in further study.

In conclusion, H19/miR-675 downregulates APC transcription, which then degrades protein complex, directly drives β-catenin transferring into nuclear and activates the Wnt/β-catenin pathway. We elucidated the trans-regulatory function of “lncRNA-H19/miR-675/APC/ Wnt/β-catenin axis” in promoting HAMSCs-droved osteogenesis both *in vitro* and *in vivo*. Based on these results, we might attach HAMSCs^H19^ into scaffold biomaterials, such as gel or nanofiber, and implant them in the bone defects around dental implants to enhance osteogenesis of existing HBMSCs. Our findings highlight the application of lncRNA-H19 and HAMSCs in the field of bone regenerative medicine and propose a paramount therapeutic target for bone deficiency.

## MATERIALS AND METHODS

### Cell culture

The Ethics and Research Committee of Nanjing Medical University approved the study protocols (Permit Number: 2018-190). Informed consent was obtained from all the participants. HAMSCs were collected from discarded amniotic membrane samples using the pancreatin/collagenase digestion method as previously described [52]. Mandible samples were collected from eight patients aged 20-30 years undergoing Sagittal Split Ramus Osteotomy (SSRO) at the Department of Oral and Maxillofacial Surgery, Affiliated Hospital of Stomatology, Nanjing Medical University. HBMSCs were collected from these samples following a previously described approach [53]. Isolated cells were maintained in Dulbecco’s Modified Eagle Medium (DMEM) (HyClone Laboratories Inc., Logan, UT, USA) supplemented with 15% fetal bovine serum (FBS), 100 U/L penicillin and 100 mg/L streptomycin (Gibco; Thermo Fisher Scientific, Inc., Waltham, MA, USA) in a humidified atmosphere containing 5%CO_2_/95% air at 37ºC. Culture medium was replaced every other day; 3-6 passages cells were harvested for the subsequent experiments.

### Co-culture system

HBMSCs and HAMSCs were seeded at an initial cell density of 5 × 10^4^ cells/cm^2^ in 6-well culture plates (Millipore®, Bedford, MA, USA) and in transwells (6-Well Millicell Hanging Cell Culture Inserts, 0.4 μm, PET, Millipore®, Bedford, MA, USA), respectively. After the cells were attached, transwells containing HAMSCs were moved into the corresponding wells containing HBMSCs to create the HAMSCs/HBMSCs coculture system.

### Lentivirus infection

Recombinant lentiviruses containing full-length H19 (Gene Bank accession number, NR_002196.1) and scramble control (NC) were obtained from Integrated Biotech Solutions Company (Shanghai, China) and were named Lenti-H19 and Lenti-NC. Recombinant lentiviruses targeting H19 and scramble control named Lenti-shH19 and Lenti-shNC, respectively, were obtained from GenePharma Company (Shanghai, China). All lentivirus vectors contained the green fluorescent reporter gene (GFP). The viruses were used to infect HAMSCs and establish stably expressing transfectants. HAMSCs were exposed to viral supernatant containing 1 mL DMEM supplemented with 10% FBS and 8 μg /mL polybrene (POL) for 10 h.

### Transfection of miRNA mimics/ inhibitors

MiRNA plasmids were obtained from Ribobio Company (Guangzhou, China). Transfection of HAMSCs with the miRNA duplexes was carried out with transfection reagent riboFECT^TM^ CP (Ribobio, Guangzhou, China). The mutated binding sites of miR-675 in luciferase reporter vectors containing APC were constructed by site-directed mutagenesis. Transient transfection was conducted using Lipofectamine 2000^®^ (Invitrogen; Thermo Fisher Scientific, Inc.) according to the manufacturer's protocol.

### Proliferation assay

The proliferation level of HBMSCs was performed using a FACScan flow cytometer (BD Biosciences, Franklin Lakes, NJ, USA) as previously described [54]. Cell cycle fractions (G0, G1, S, and G2 M phases) were processed and analyzed using MODFIT LT 3.2 (Verity Software House, Topsham, ME, USA).

### Alkaline phosphatase (ALP) staining and activity assay

Osteogenic differentiation was induced using osteogenic media containing 100 nM dexamethasone, 10 mM β-glycerophosphate and 100 nM ascorbic acid (all Sigma Chemical Co., St. Louis, MO, USA) for 7 days. ALP staining was detected using NBT/BCIP staining kit (CoWin Biotech, Beijing, China) as previously described [55]. ALP activity was measured using an ALP assay kit (Jiancheng Corp, Nanjing, China) based on the absorbance at 405 nm [56]. The total protein content of each sample was determined with a BCA kit (Beyotime, China). The enzyme activity was expressed as micromoles of reaction product per minute per total protein.

### Alizarin red staining and quantification

Osteogenic differentiation was induced for 14 days. Mineralized matrix formation was determined by 40 mM/L alizarin red (pH = 4.2, Sigma Chemical Co., St. Louis, MO, USA) at room temperature for 20 min. Alizarin red quantification was performed based on the absorbance at 570 nm. The final calcium concentration was normalized to the total protein content.

### RNA Isolation and quantitative real-time reverse transcription polymerase chain reaction (RT-PCR)

Cellular RNA was isolated using TRIzol reagent (Invitrogen, New York, NY, USA) according to the manufacturer's protocol, and then reversely transcribed into cDNA using the Reverse Transcription Kit (Applied Biosystems, Foster City, CA). RT-PCR was conducted with SYBR Green Master (Roche, Indianapolis, IN, USA) and ABI Prism 7500 real-time PCR System (Applied Biosystems). The following thermal settings were performed: 95°C for 10 minutes followed by 40 cycles of 95°C for 15 seconds and 60°C for 1 minute. Primers used in this study are listed in Table 1. Human U6 RNA was applied as an internal control for miRNA, and human GAPDH was used as a control for normalizing expressions of lncRNA and mRNAs. The data were calculated using the 2^−ΔΔCt^ method.

**Table 1:** Primers used for quantitative real-time reverse transcription polymerase chain reaction.

**Genes**	**Sense primer(5’-3’)**	**Anti- sense primer(5’-3’)**
ALP	AGAACCCAAAGGCTTCTTC	CTTGGCTTTTCCTTCATGGT
RUNX2	TCTTAGAACAAATTCTGCCCTTT	TGCTTTGGTCTTGAAATCACA
OCN	AGCAAAGGTGCAGCCTTTGT	GCGCCTGGGTCTCTTCACT
OSX	CCTCCTCAGCTCACCTTCTC	GTTGGGAGCCCAAATAGAAA
H19	CTTTCATGTTGTGGGTTCTGG	CGGGTCTGTTTCTTTACTTCC
APC	AAAGTGAGCAGCTACCACG	CCTGGAGTGATCTGTTAGTCG
*β*-Catenin	AGCTGACAACTTTCACACC	AATGGGGATGTTGATCTTC

### Western blot

Western blot analysis was performed as previously described [57]. The primary antibodies were as follows: RUNX2 (D1L7F) Rabbit mAb #12556(1:1000), APC Antibody #2504(1:1000), *β*-Catenin (D10A8) XP® Rabbit mAb #8480(1:1000), Cyclin D1 (92G2) Rabbit mAb #2978(1:1000), c-Myc (E5Q6W) Rabbit mAb #18583(1:1000), *β*-actin (8H10D10) Mouse mAb #3700(1:1000) (All from Cell Signaling Technology, Danvers, MA, USA), anti-ALP (ab83259) (1:1000), anti-osteocalcin (OCN) (ab133612) (1:1000), anti- Osterix (OSX) (ab209484) (1:1000) (All from Abcam, Cambridge, MA, USA). *β*-actin served as an internal control. Western blot analysis was quantified using ImageJ software (http://rsb.info.nih.gov/ij/) and the signal of each target band was normalized to that of the *β*-actin band.

### Immunofluorescence staining

Transfected HAMSCs grown on 10 mm^2^ glass coverslips were fixed with 4% paraformaldehyde for 30 minutes at room temperature, permeabilized with 0.1% Triton X-100 for 12 minutes, and then blocked with 3% bovine serum albumin (BSA; Sigma-Aldrich) for 45 min at 37 °C. Thereafter, primary antibody [*β*-Catenin (D10A8) XP® Rabbit mAb #8480(1:100), Cell Signaling Technology, Danvers, MA, USA] were incubated and conducted at 4 °C overnight, followed by specified secondary antibody labeling for 30 min at 37 °C in dark. Nuclei were counterstained with DAPI. Images were observed with the inverted fluorescence microscope (Olympus, Japan).

### Dual luciferase reporter assay

Luciferase assays were performed as previously described [58]. Briefly, the HEK293T cells cultured in a 24-well plate were transfected with luciferase plasmids and miR-675 mimic or negative control using Lipofectamine 2000. Luciferase activities were measured 48 hours after transfection using Dual Luciferase Reporter Assay System (Promega).

### In vivo critical-sized mandibular defect model

A total of 16 female nude rats (RNU, Charles River, Wilmington, MA), with an average weight of 280g, were obtained from Nanjing Medical University. All the animals were housed in an environment with a temperature of 22 ± 1 ºC, a relative humidity of 50 ± 1% and a light/dark cycle of 12/12 hr. All animal studies (including the mice euthanasia procedure) were done in compliance with the regulations and guidelines of Nanjing Medical University institutional animal care and conducted according to the AAALAC and the IACUC guidelines. Under general anesthesia, a critical-size mandible defect (5x5 mm) was made using a 5mm stainless steel tissue punch. Approximately 10 × 10^4^ cells (5x10^4^ HAMSCs or HAMSCs ^shH19^ and 5x10^4^ HBMSCs) were attached to each HA/TCP biomaterial (Φ5×H2mm, Sichuan University, Chengdu, Sichuan, China). After 12 hours, the complexes were subcutaneously implanted into the mandibular defect area. Animals were then randomly allocated into cages; 3–4 animals were housed per cage in standard cages at 25°C. In addition, animals had free access to rodent chow and water.

### 3D micro-computerized tomography (micro-CT) scanning

Eight weeks after implantation, animals were sacrificed and mandibles were harvested for micro-CT analysis. The high-resolution micro-CT machine (Scanco USA, Inc., Southeastern, PA), Dolphin 3D software (Dolphin Imaging & Management Solutions, Chatsworth, CA) and CTAn (Skyscan, Kontich, Belgium) were used. For examining the bone structure, the bone volume ratio (BV/ TV, %) was calculated.

### Histological observation

After micro-CT analysis, samples were harvested for histologic staining as previously described [59]. The bone matrix was analyzed by hematoxylin and eosin (H&E) and Masson trichrome. For immunohistochemistry, decalcified sections were blocked with goat serum, incubated with primary antibodies against RUNX2 (1:300 dilution) at 4°C overnight, and immunohistochemical staining was captured under the microscope.

### Statistical analysis

Representative data are presented as the mean and standard deviation (SD) of at least three independent samples. *P* values < 0.05 were considered as statistically significant using one-way analysis of variance (ANOVA).

## Supplementary Material

Supplementary Figure 1
