# Supplementary material for: Human amnion-derived mesenchymal stem cells promote osteogenic differentiation of human bone marrow mesenchymal stem cells via H19/miR-675/APC axis
Source: Aging (Albany NY). 2020 May 20;12(11):10527–43. doi: 10.18632/aging.103277 (PMC7346082; doi:10.18632/aging.103277)
Supplement: Supplementary Figure 1 [file aging-12-103277-s001..pdf]

SUPPLEMENTARY FIGURE

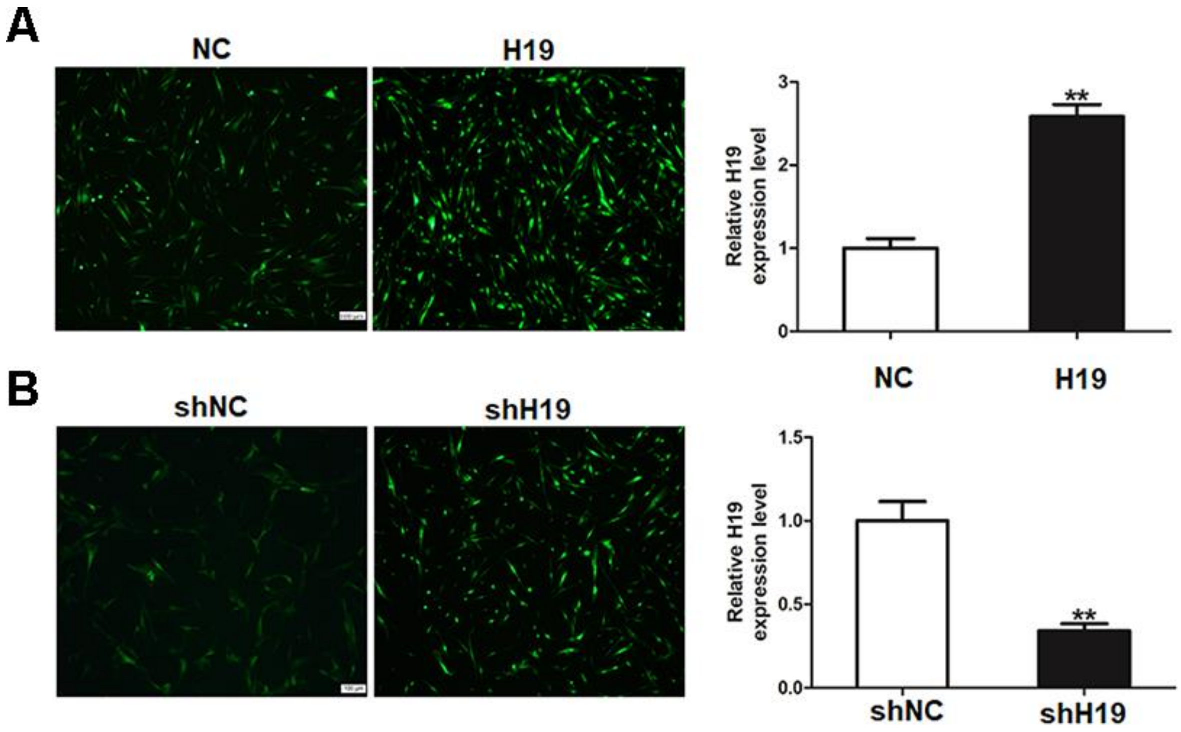

Supplementary Figure 1. Establishment of stably expressing transfectants. (**A**, **B**) Fluorescent photomicrographs and RT-PCR showed lentivirus transduction and relative H19 expression in NC, H19, shNC, and shH19 groups. Scale bar, 100 μm. Data are shown as mean ± SD.**$P < 0.01$.
